# Bioinformatics and Experimental Analyses Reveal MAP4K4 as a Potential Marker for Gastric Cancer

**DOI:** 10.3390/genes13101786

**Published:** 2022-10-03

**Authors:** Junping Zhang, Xiaoping Cai, Weifeng Cui, Zheng Wei

**Affiliations:** 1 Cancer Research Institute, Henan Academy Institute of Chinese Medicine, Zhengzhou 450000, China; 2 School of Basic Medicine Sciences, Henan University of Chinese Medicine; Zhengzhou 450004, China

**Keywords:** gastric cancer, MAP4K4, GSEA, miRNA target, methylation

## Abstract

Background: Gastric cancer remains the most prevalent and highly lethal disease worldwide. MAP4K4, a member of Ste20, plays an important role in various pathologies, including cancer. However, its role in gastric cancer is not yet fully elucidated. Therefore, this study aims to determine the tumor-promoting role of MAP4K4 in gastric cancer and whether it can be used as a new and reliable biomarker to predict the prognosis of gastric cancer. For this purpose, we divide the samples into high- and low-expression groups according to the expression level of MAP4K4. The association of MAP4K4 expression with prognosis is assessed using the Kaplan–Meier survival analysis. Furthermore, immune infiltration analysis using ESTIMATE is conducted to evaluate the tumor immune scores of the samples. Results: The findings reveal a significantly higher expression of MAP4K4 in tumor samples than in adjacent samples. The high-expression group was significantly enriched in tumor-related pathways, such as the PI3K-Akt signaling pathway. In addition, immune infiltration analysis revealed a positive correlation between immune scores and MAP4K4 expression. We also observed that miRNAs, such as miR-192-3p (R = −0.317, *p*-value 3.111 × 10^−9^), miR-33b-5p (R= −0.238, *p*-value 1.166 × 10^−5^), and miR-582-3p (R = −0.214, *p*-value 8.430 × 10^−5^), had potential negative regulatory effects on MAP4K4. Moreover, we identified several transcription factors, ubiquitinated proteins, and interacting proteins that might regulate MAP4K4. The relationship between MAP4K4 and DNA methylation was also identified. Finally, we verified the high expression of MAP4K4 and its effect on promoting cancer. Conclusion: MAP4K4 might be closely related to gastric cancer’s progression, invasion, and metastasis. Its high expression negatively impacts the prognosis of gastric cancer patients. This suggests MAP4K4 as an important prognostic factor for gastric cancer and could be regarded as a new potential prognostic detection and therapeutic target.

## 1. Introduction

Stomach adenocarcinoma (STAD) is one of the most common gastrointestinal malignancies, ranking fifth in global cancer incidences and third in cancer mortality [1,2]. Although five-year survival rates of 90% to 97% can be achieved, the five-year survival rate in patients with metastatic or advanced-stage STAD is less than 30%, which can reach 90% to 97% with the early diagnosis and early treatment of STAD [1,2]. The malignant phenotype of STAD is not only determined by the intrinsic activity of cancer cells, but also by the recruited and activated stromal and immune cells in the tumor-associated microenvironment, which are major components necessary for tumor development and progression [3]. Accumulating evidence suggests that the tumor microenvironment (TME) plays a key role in the occurrence, progression, prognosis, and response to immunotherapy of STAD [4,5]. The stromal cells and their interaction with tumor cells contribute to tumor progression, invasion, and spread [6]. STAD is chronic gastritis caused by *Helicobacter pylori* and is often characterized by immune cell infiltration, including granulocytes, macrophages, and T lymphocytes [7,8]. Tumor-associated lymphocytes (TALs), mainly T cells, are the main infiltrating immune cell type in cancer tissues and produce soluble cytokines. These soluble cytokines can regulate cancer cell proliferation and migration, promote angiogenesis, and are involved in activating host defense mechanisms [9,10,11]. In STAD, tumor tissue infiltration caused by tumor-associated macrophages (TAMs) is significantly correlated with tumor angiogenesis, tumor invasion depth, lymph node status, and clinical stage [12,13]. Furthermore, TME alteration may contribute to the development of STAD heterogeneity through the extrinsic activation of various genes and signaling pathways. In addition, stromal cells can secrete growth factors, cytokines, and chemokines, which are critical for tumor characteristics [14]. Therefore, it is crucial to explore new and reliable biomarkers to predict the prognosis of STAD.

The MAP4K4 (mitogen-activated protein kinase Kinase Kinase Kinase 4), also known as HGK (hematopoietic progenitor kinase/germinal center kinase-like kinase), is a serine/threonine kinase belonging to the Ste20 kinase family [15,16]. The role of MAP4K4 in immune, inflammatory, metabolic, and cardiovascular diseases has been recognized [17,18,19]. The knowledge of MAP4K4 in cancer is minimal, but increasing evidence suggests its role in cancer [20,21,22,23] and might act as a new actionable target for cancer therapy. It has been suggested that MAP4K4 can promote oncogenic transformation by regulating its downstream targets [24]. However, information on the regulation of MAP4K4 by natural stimuli is still limited. It has been reported that MAP4K4 possibly plays a role in response to environmental stress and cytokines, such as TNF-α. TNF-α has been reported to be a bona fide agonist of MAP4K4, and TNF-α is known to induce inflammation and cancer [25,26]. To our knowledge, no study reports the role of MAP4K4 in gastric cancer, so whether and how MAP4K4 plays a role in gastric cancer still needs to be further explored. Moreover, a quaternary benzophenanthridine alkaloid extracted from plant (Papaveraceae) roots has shown significant anti-tumor activity against multiple cancer cells [27]. Therefore, further research is needed to ensure that all patients with cancer receive safe and effective therapies and the highest possible quality of life.

Expression profiling and prognostic analysis are performed based on mRNA expression and clinical data of gastric cancer patients from public datasets. Functional analysis is conducted for the identified differentially expressed genes between high- and low-expression groups of MAP4K4. In addition, we also compare and analyze the immune microenvironment of MAP4K4 groupings in gastric cancer, and the results show that some immune scores are positively correlated with MAP4K4. Finally, we confirm the high expression of MAP4K4 in gastric cancer and its possible carcinogenic effect. These results might provide new insights into the targets and methods for gastric cancer treatment.

## 2. Methods

### 2.1. Data Source and Preprocessing

The TCGA-LIHC dataset (hereafter referred to as the TCGA dataset) sourced from The Cancer Genome Atlas (TCGA) database was downloaded from UCSC Xena (https://xenabrowser.net/, accessed on 20 February 2021), including RNA sequencing (RNA-seq) data (FPKM format), somatic mutation data, and clinical information of 375 gastric cancer and 294 normal samples. For RNA-seq data, the edgeR package of R language [28] was used to convert the gene counts data based on the qCML method for CPM (counts per million reads), then CPM was converted to log_2_ (CPM + 1). *p*-values were calculated based on negative binomial distributions combined with Fisher’s exact. The somatic mutation data included single nucleotide variations (SNVs) and copy number variations (CNVs). The SNV data were processed by mutect, and CNV data were processed by GISTIC algorithm as previously described [29]. Methylation data were downloaded from the LinkedOmics database (http://www.linkedomics.org/login.php, accessed on 24 February 2021). In addition, tumor mutational burden (TMB) data were downloaded.

### 2.2. Construction of Survival Model

First, the survival package [30] of R language was used to calculate the HR value of the Cox risk regression model and the chi-squared test *p*-value for the survival of the high- and low-expression groups based on the TCGA data set. TCGA clinical data were downloaded from the Genomic Data Commons data portal (GDC http://gdc-portal.nci.nih.gov/legacy-archive/, accessed on 24 February 2021) [31]. Based on the gene expression median values, the corresponding cancer samples were divided into two groups of high and low expressions. The genes with expressions higher than median values belonged to the high-expression group, whereas the genes with expressions lower values than median values were assigned to the low-expression group. Then, the Kaplan–Meier survival analysis was performed using the corresponding clinical data. Finally, the disease-free and overall survival curves of the high- and low-gene-expression groups were illustrated.

In addition, univariate Cox regression analysis was performed on gene expression and other clinical data using the survival package [30] of Rstudios. The corresponding cancer samples were divided into two groups of high and low expressions based on the median gene expression. The chi-squared test was performed on the clinical data to explore if the sub-classification of various clinical data was related to the distinction between high- and low-gene-expression samples.

### 2.3. Tumor Immune Microenvironment Analysis

R package ESTIMATE (Estimation of Stromal and Immune cells in MAlignant Tumor tissues using Expression data) [32] was used to evaluate the tumor immune microenvironment scores, and the differential distribution in different groups was compared. Correlational analyses were conducted between the expression of individual genes and these immune infiltration values.

In addition, based on the gene expression feature set of 22 immune cell subtypes by default, CIBERSORT [33] was used to deconvolute the expression matrix of human immune cell subtypes based on the principle of linear support vector regression.

### 2.4. miRNA and Transcription Factor Target Analysis

For MAP4K4, the miRNA regulatory relationship was predicted in five miRNA regulatory databases: miRDB [34], mirtarbase [35], miRMap [36], miRanda [37], and TargetScan [38]. In addition, the transcription factors that could regulate MAP4K4 were predicted based on the ENCODE database [39].

### 2.5. Protein Ubiquitination Analysis

UbiBrowser [40] is an integrated bioinformatics platform for predicting and presenting a naive Bayesian network-based network of whole-proteome human E3 substrates. In the present study, UbiBrowser was used to predict the potential protein ubiquitination of MAP4K4.

### 2.6. Protein Interaction Network Prediction

The STRING database is used for known and predicted protein–protein interactions (PPIs) [41]. PPI includes direct (physical) and indirect (functional) associations, which arise from computational predictions, knowledge transfer between organisms, and interactions aggregated from other (primary) databases. Based on the protein interaction relationship in the STRING database, a screening interaction score ≥ 0.400 (medium confidence) was set as the threshold to screen potential PPIs of MAP4K4.

### 2.7. Gene-Set-Enrichment Analysis (GSEA) and Functional Annotation

GSEA [42] was used to perform pathway analysis. All candidates in the the Kyoto Encyclopedia of Genes and Genomes (KEGG) pathway provided by MSigDB [43] were used in the GSEA. The GSEA input file contained the expression profile data and sample labels (high-or low-gene-expression groups). Furthermore, KEGG pathway analysis and gene ontology (GO) functional-enrichment analysis were performed on the differential genes grouped by LIHC through the WebGestaltR (v0.4.2) package [44] of R language.

### 2.8. Patients and Clinical Specimens

Written informed consent was signed by each patient. At the same time, all aspects of this study were approved by the hospital research ethics committee. Fresh specimens from 21 gastric cancers were collected, and the compared surrounding tissues were used as controls. All patients received no anticancer drug treatment, chemotherapy, radiotherapy, or biological immunotherapy before surgery. All samples were confirmed by a laboratory pathological examination. The clinical information of these patients is presented in Table 1.

### 2.9. Cell Culture

The cell lines, GES-1, MGC-803, MKN-28, SGC-7901, and OCUM-1, were purchased from the Type Culture Collection of the Chinese Academy of Sciences (Shanghai, China). The cell lines, GES-1, MKN-28, SGC-7901, and OCUM-1 were cultured, respectively, in RPMI-1640 medium (Gibco; Thermo Fisher Scientific, Inc., Waltham, MA, USA) supplemented with 10% FBS (Gibco; Thermo Fisher Scientific, Inc.) at 37 °C with 5% CO_2_. MGC-803 cells were cultured in DMEM medium supplemented with 10% FBS (Gibco; Thermo Fisher Scientific, Inc.) at 37 °C with 5% CO_2_.

### 2.10. Vector Construction and Transfection

Two shRNA sequences of the MAP4K4 gene with a shRNA control sequence were designed and constructed into a pLVX-shRNA2-Puro lentivirus vector. After the successful construction of the vectors was verified by sequencing, the vectors were transfected to 293T cells. After 12 h of transfection, the cell culture medium was replaced with a virus packaging solution containing 2% fetal bovine serum. After 48 h of continuous culture, the cell supernatant was collected. After being filtered with a 0.45 μm filter, gastric cancer cells MKN-28 and SGC-7901 were infected with the lentivirus, respectively, and then the supernatant was exchanged for fresh medium for 12 h with 3 μg/mL of puromycin. After obtaining stable cell lines, cell RNA and protein were collected, the knockdown efficiency of which was verified by detecting the expression levels of MAP4K4 mRNA and protein, and the best knockdown efficiency of the shRNA sequence was selected for subsequent experimental research.

### 2.11. Real-Time Quantitative PCR

Total RNA (tRNA) was isolated from cell lines using the RNeasy Mini Kit (Cat: 74104) (QIAGEN, Hilden, Germany). Reverse transcription PCR (RT-PCR) was performed using Superscript IV Reverse Transcriptase (Cat: 18090010) (ThermoFisher, Waltham, MA, USA). Triplicate samples and their corresponding controls were evaluated by qPCR. The 2-ΔΔCt algorithm determined gene fold changes. Human β-actin (1:500, ab189073, abcam) was used as a reference gene to normalize 2-ΔΔCt-based assessments, and GES-1 was used to normalize expressions between cell lines. The relative expression of genes was normalized to human β-actin. All primer sequences used are listed in Table 2.

### 2.12. Transwell Invasion Assay

For this assay, 24-well Transwell chambers (3422, Corning) were utilized. Briefly, gastric cancer cells of “10^5^” cells/well were grown on the top of the incubator using a Matrigel-containing (250 μg/mL) serum-free medium. A total of 600 μL of complete medium (enriched culture medium to contain all the growth requirements) was added to the bottom space. After 24–48 h of culture, the cells on the upper surface of the apical cavity were removed with a cotton swab, and the infiltrating cells on the lower surface of the apical cavity were stained with Harris hematoxylin solution (Cat: HHS16) (Sigma-Aldrich, USA) and examined under a microscope.

### 2.13. Wound-Healing Assay

Gastric cancer cells, whose fusion rate was 100%, were cultured in six-well plates. Then, these cells were starved with sterile 200 μL pipette tips to an injury state, then incubated for 24 h after washing. In addition, light microscope observation was performed 24 h before and after treatment. A digital camera was used to observe, measure, and photograph the wound healing of each group.

### 2.14. Clone Formation Assay

Cells were trypsinized into a single-cell suspension at a density of 250 cells/mL through the treatment with 0.25% trypsin. Then, these cells were seeded in a 6-well plate (500 cells per well). After culture for 14 days at 37 °C in a humidified environment with 5% CO_2_, the cells were fixed in absolute ethanol for 10 min and then stained with 0.1% crystal violet for 1/2 an hour. Colonies of more than 50 cells were counted, and relative colony numbers were obtained. Colony-forming ability was assessed by comparing the number of colonies formed in the transfected cells to the number of colonies formed in the control group.

### 2.15. Western Blot

RIPA lysis buffer (Beyotime Biotechnology, Shanghai, China) was used for protein extraction from gastric gland tissue. SDS-PAGE was used to separate protein samples (60 μg) which were then transferred to polyvinylidene difluoride (PVDF) membranes (microwells). The protein was incubated on the membrane overnight at 4 °C after blocking using the primary antibody (MAP4K4 antibody, 1:1000; (Cat: ab155583); Abcam, Cambridge, MA, USA). Then, HRP-conjugated secondary antibodies (Cat: R-05073-500) were used for the incubation the next day. In addition, a fluorescence imager (α) was used for visualization, and specific proteins’ expression levels were normalized to β-actin levels.

### 2.16. Statistical Analysis

All statistical analyses and visualizations were performed using Microsoft Excel, GraphPad Prism 8.0, and RStudio software (version 4.1.0), except as otherwise noted. Clinical characteristics were expressed as mean ± standard deviation or *n* (%). Multivariate partial-least-squares discriminant analysis (PLS-DA) with 200 permutation tests was performed using SIMCA-P 14.1 (Umetrics, Umea, Sweden). For univariate analysis, the Wilcoxon signed-rank test was performed on matched samples using MATLAB software (R2014a, MathWorks, Natick, MA, USA). The Benjamini–Hochberg method was used to control the false discovery rate (FDR). Spearman correlation analysis was performed to determine correlations between the identified features and clinical parameters. Using the Student’s paired t-test, the expression of MAP4K4 in tissues was compared. *p*-value < 0.05 was considered statistically significant.

## 3. Results

### 3.1. MAP4K4 Expression Was Correlated with STAD Overall Survival

**Figure 1:**
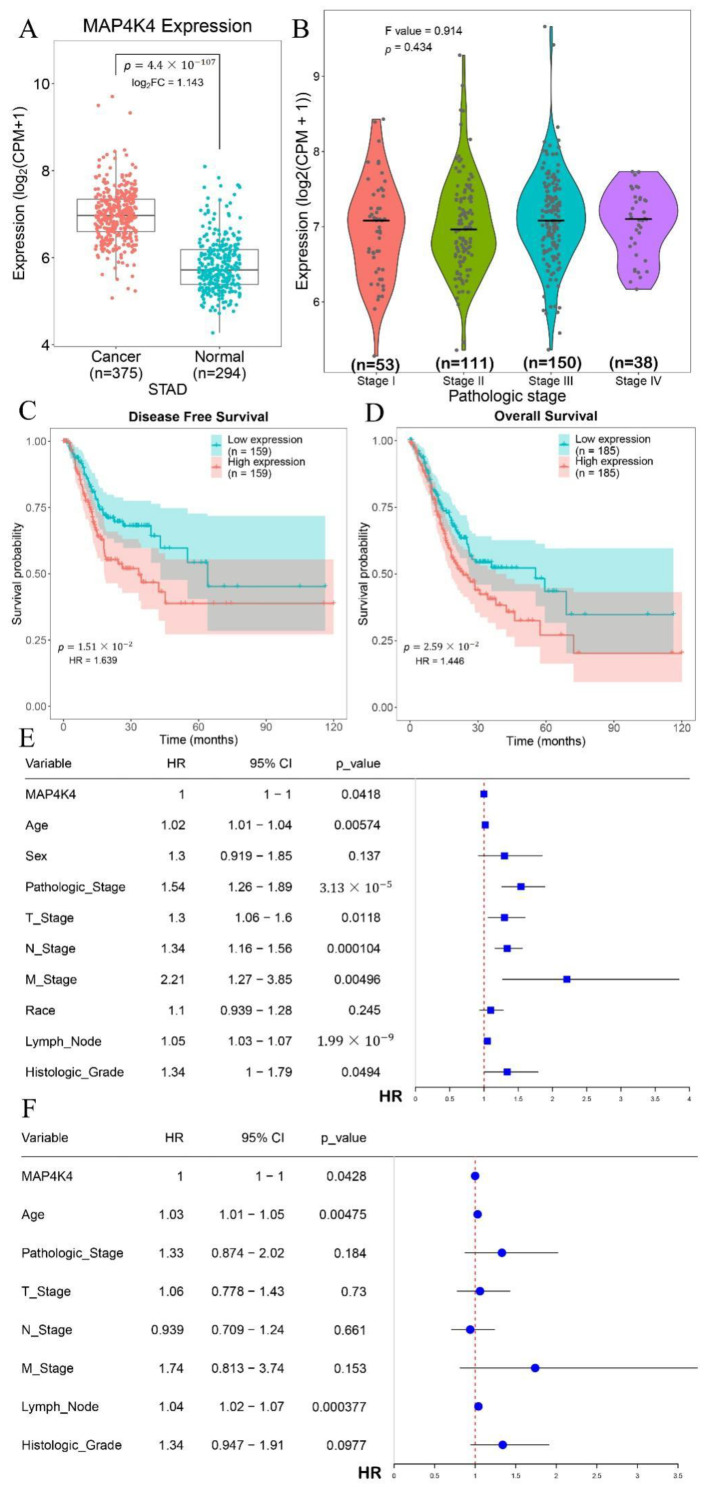
**Differentially expressed MAP4K4 and survival differences.** (**A**) Differential expression of MAP4K4 in gastric cancer and normal samples; (**B**) the expression of MAP4K4 in samples of different pathological stages; (**C**) the disease-free survival curve of high- and low-MAP4K4-expression groups; (**D**) the overall survival curve of high- and low-MAP4K4-expression groups; HR, hazard ratio of survival, and *p*-value was used to check the survival significance between high- and low-MAP4K4-expression groups, whereas the cl values represent the confidence levels; (**E**) the results of multivariate Cox regression analysis; (**F**) the results of univariate Cox regression analysis. The lines in plot represent 95% CI of HR.

The gene expression levels of MAP4K4 in tumor and normal (tumor-adjacent) samples were first assessed. The expression of MAP4K4 in tumor samples was significantly higher than in normal samples (Figure 1A, *p*-value 4.4 × 10^−107^), suggesting that MAP4K4 might play an oncogenic role in gastric cancer. In addition, the expression of MAP4K4 in samples of different pathological stages revealed no significant difference in the expression of MAP4K4 among the four stages, stages I, II, III, and IV (Figure 1B, *p*-value 0.434), indicating that MAP4K4 was overexpressed in all four stages.

To detect whether MAP4K4 expression was related to overall survival, the cancer samples were divided into two groups with high and low expressions based on the median expression of MAP4K4 using the survival package in R language. Kaplan–Meier survival analysis showed that the disease-free (Figure 1C, *p*-value 1.51 × 10^−2^) and overall (Figure 1D, *p*-value 2.59 × 10^−2^) survival were significantly different between the two groups. Groups with a higher MAP4K4 expression were positively correlated with a poorer prognosis, suggesting the MAP4K4 gene’s association with survival and poor prognosis. Moreover, the group with a higher MAP4K4 expression presented a higher risk, indicating that MAP4K4 is a risk factor in gastric cancer.

In addition, univariate Cox regression analysis was employed to analyze the HR, 95% CI of HR, *p*-value for the association between clinical features, and MAP4K4 expression (Figure 1E). The clinical information of TCGA patient records, including age, sex, pathologic stage, T stage, N stage, M stage, race, lymph node, and histologic grade, were systematically analyzed. Among these factors, M stage had the highest risk (HR = 2.21, 95% CI = 1.27–3.85, *p*-value 0.00496), followed by pathologic stage (HR = 1.54, 95% CI = 1.54, *p*-value 3.13 × 10^−5^). In the multivariate Cox regression analysis of MAP4K4, the M stage presented the highest risk (HR = 1.74, 95% CI = 0.813–3.74, *p*-value 0.153 (Figure 1F).

Furthermore, the corresponding cancer samples were divided into two groups with high- and low-MAP4K4 expressions based on the median value of the MAP4K4 expression, and then the chi-squared test was performed on the clinical data to analyze whether the sub-classification of various clinical data was related to the distinction between high- and low-MAP4K4-expression samples (Table 3). The findings revealed that various clinical data subcategories are not significantly correlated with MAP4K4 expression.

Table 3 mainly describes the characteristics where high and low expressions are described. However, their significant variation was measured by the *p*-value. No significant differences were observed for age, sex, and pathologic stage. Table 3 was statistically analyzed using the chi-squared test.

### 3.2. The Relationship between MAP4K4 Expression and Cellular Immune Infiltration

**Figure 2:**
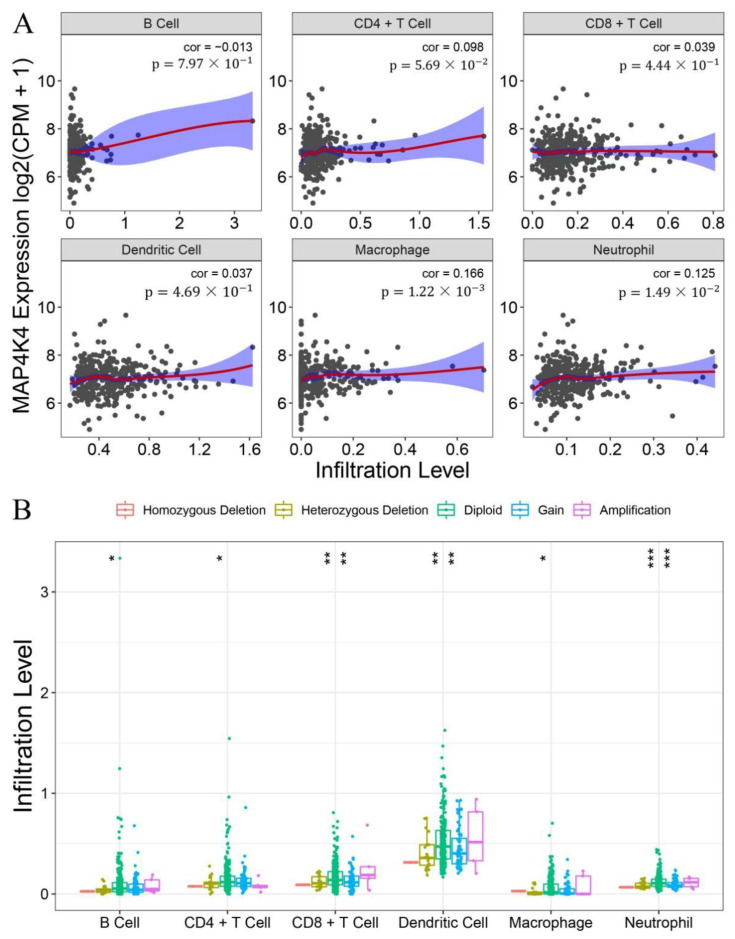
Statistics of cellular immune infiltration and corresponding copy number variation data in corresponding diseases. (**A**) the Spearman correlation between MAP4K4 expression and the immune infiltration level of 6 types of immune cells; (**B**) the CNV classification of MAP4K4 in the immune infiltration level of 6 types of immune cells. Herein, in Figure 2B, * represents the significance level that is analyzed using multivariant analysis. (* FDR < 0.05, ** FDR < 0.01, *** FDR < 0.001).

Subsequently, we analyzed the correlation between MAP4K4 expression and the level of immune cell infiltration, including B cells, CD4+ T cells, CD8+ T cells, dendritic cells, macrophages, and neutrophils. The results show that the expression of MAP4K4 has a weak positive correlation with macrophages (cor = 0.16, *p*-value 1.22 × 10^−3^) and neutrophils (cor = 0.125, *p*-value 1.49 × 10^−2^) (Figure 2A). Mutations, such as the amplification and deletion of MAP4K4, were also examined. These mutations mainly included amplification (two or more extra copies), acquired mutations (one extra copy), diploidy (normal copy number), heterozygous deletion (one missing copy), and homozygous deletion (two missing copies). Immune infiltration levels of MAP4K4 mutant phenotypes were examined in the abovementioned six types of immune cells. The finding reveal that the diploidy represents the highest infiltration level in all these six types of immune cells, indicating that the CNV mutation of MAP4K4 significantly correlates with the lower level of immune infiltration.

### 3.3. Potential Regulatory Mechanisms of MAP4K4

**Figure 3:**
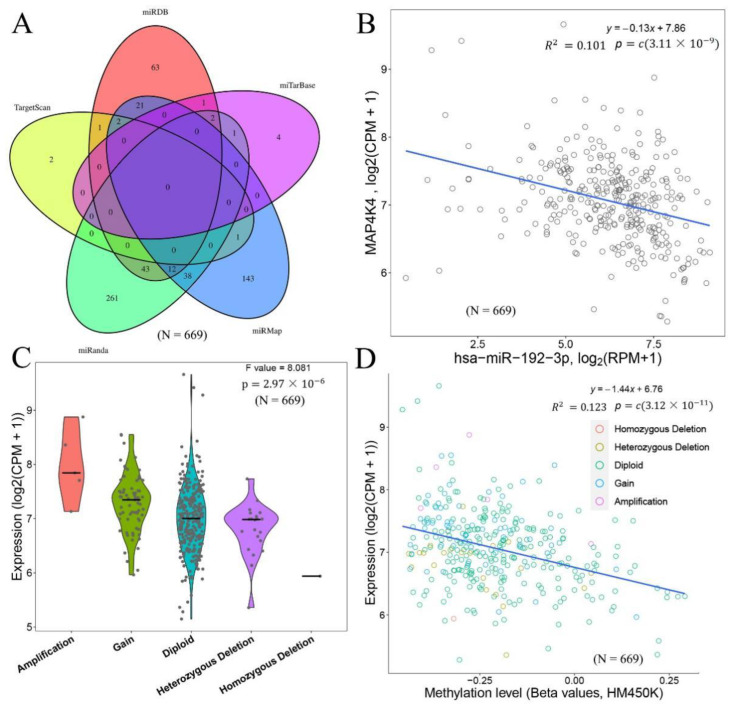
miRNA regulation relationship and CNV correlation analysis of MAP4K4. (**A**) Venn diagram of the predicted miRNA regulatory relationship with MAP4K4 in 5 miRNA databases: miRDB, miTarBase, miRMap, miRanda, and TargetScan; (**B**) correlation diagram between MAP4K4 and has-miR-192-3p; (**C**) the expression of MAP4K4 in various types of CNVs in the cancer sample; (**D**) scatter plot and correlation of MAP4K4 expression in cancer samples and the β value of the corresponding methylation level. Circles with different colors represents different CNV classifications. Infinium methylation assay, a probe-based methylation profile, is used for the methylation profile. N represents the sample size.

To explore the potential regulatory mechanisms of MAP4K4, different miRNA target gene databases were used to predict the miRNAs that might regulate MAP4K4. The predicted miRNAs were integrated to obtain common miRNAs from different databases (Figure 3A). Among them, no miRNAs were simultaneously predicted by 5 or 4 databases, 16 miRNAs were predicted by 3 databases, and 106 miRNAs were simultaneously predicted by 2 databases. The correlation between MAP4K4 and predicted miRNAs were calculated to search for regulatory miRNAs with negative correlations. These miRNAs included hsa-miR-192-3p obtained via the miRanda and miRMap databases (Figure 3B), R= −0.317, *p*-value 3.111 × 10^−9^), hsa-miR-33b-5p (R= −0.238, *p*-value 1.166 × 10^−5^) obtained via the miRDB, miRanda and miRMap databases, hsa-miR-582-3p (R= −0.229, *p*-value 1.12973) obtained via the miRDB, miRanda and miRMap databases (R= −0.214, *p*-value 8.430 × 10^−5^), hsa-miR-141-3p (R= −0.142, *p*-value 9.304 × 10^−3^), and hsa-miR-141 (R= −0.148, *p*-value 2.894) obtained via the miRDB database 181c-5p, etc.

Based on the ENCODE database, the transcription factors regulating MAP4K4 were predicted to be ARID3A, BACH1, BHLHE40, BRCA1, CBX3, CCNT2, CEBPB, CEBPD, FOS, FOXA1, FOXA2, FOXP2, and GABPA. The expression of MAP4K4 in the CNV-type Amplification, Gain, and Diploid were also presented, which showed that the type of CNV with more copies tended to have higher expression levels of MAP4K4 (Figure 3C).

**Figure 4:**
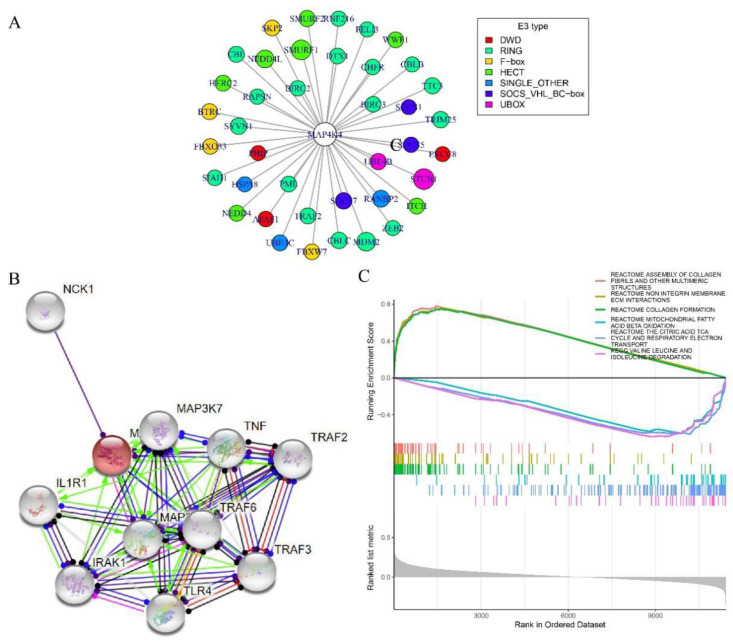
Protein and GSEA analyses of MAP4K4. (**A**) MAP4K4 protein-ubiquitination-prediction-relationship network; (**B**) MAP4K4 protein-interaction-relationship network; (**C**) GSEA analysis results of high- and low-MAP4K4-expression groups. Their statistical analysis is performed using the Cox risk regression model and chi-squared test.

In addition, the relationship between MAP4K4 expression and DNA methylation level was examined. Regardless of the CNV classification, MAP4K4 expression was negatively correlated with DNA methylation level (Figure 3D). Ubiquitination played an important role in protein localization, metabolism, function, regulation, and degradation, so the E3-type ubiquitination range of the MAP4K4 protein from UbiBrowser was collected, including SKP2, BTRC, FBXO33, and FBXW7 with F-box domain; UBE4B and STUB1 with UBOX domains; PHIP, ERCC8, and APAF1 with DWD domains; and other proteins with domains, such as RING and HECT (Figure 4A). The PPI network revealed that MAP4K4 might interact with proteins, such as IL1R1, IRAK1, MAP3K1, MAP3K7, NCK1, TNF, TRAF2, TRAF3, TRAF6, and TLR4 (Figure 4B).

The significantly enriched pathways in the high- and low-MAP4K4-expression groups were analyzed using GSEA. In the TCGA data set, collagen-related pathways, such as the reactome assembly of collagen fibrils and other multimeric structures, reactome non-integrin membrane ECM interactions, and reactome collagen formation, were significantly enriched in the high-MAP4K4-expression group. Electron transport and amino acid degradation-related pathways, such as reactome mitochondrial fatty acid β-oxidation; the citric acid TCA cycle; and respiratory electron transport, valine leucine, and isoleucine degradation were significantly enriched in the low-MAP4K4-expression group (Figure 4C).

### 3.4. Experimental Verification of the Role of MAP4K4 in Promoting Cancer in Gastric Cancer

**Figure 5:**
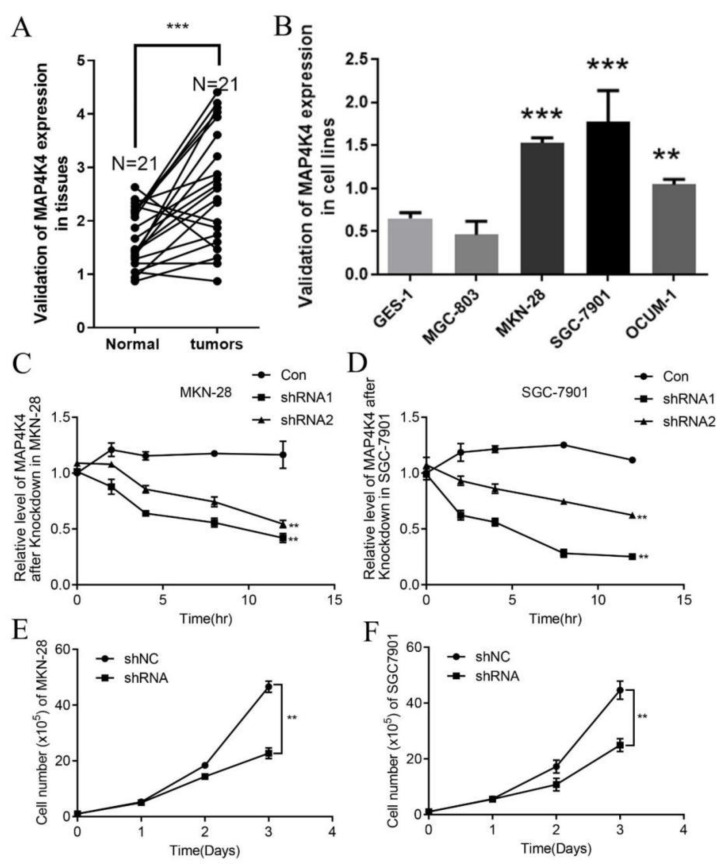
Detection of MAP4K4 expression changes. (**A**) q-PCR detection of MAP4K4 expression in gastric cancer (*n* = 21) and adjacent samples (*n* = 21), and the significance is conducted by Student’s paired t-test; (**B**) q-PCR detection of MAP4K4 expression of gastric cancer cell lines (MGC-803, MKN-28, SGC-7901, and OCUM-1) and control cells (human gastric mucosa epithelial cell line GES-1); (**C**) relative expression level of MAP4K4 after 0 h, 2 h, 4 h, 8 h, and 12 h of MAP4K4 silencing in MKN-28 cells; (**D**) relative expression level of MAP4K4 after 0 h, 2 h, 4 h, 8 h, and 12 h of MAP4K4 silencing in SGC-7901 cells; (**E**) cell number of MKN-28; (**F**) cell number of SGC-7901 that represent the significant correlation between cell numbers and time interval. A total of 375 gastric cancer and 294 normal samples are checked for statistical variation and calculated using Fisher’s exact test. Data are derived from independently repeated experiments (Mean ± SD). ** *p* < 0.01, *** *p* < 0.001.

To validate the cancer-promoting role of MAP4K4 in gastric cancer, we first detected the differences in MAP4K4 expressions in tumor and adjacent samples. MAP4K4 was upregulated in tumor samples compared with adjacent samples (Figure 5A). Meanwhile, the expression of MAP4K4 was determined by qPCR in gastric cancer cell lines (MGC-803, MKN-28, SGC-7901, and OCUM-1) and control cells (human gastric mucosa epithelial cell GES-1 cell line). The expressions of MAP4K4 were highest in SGC-7901 and MKN-28, while the lowest in MGC-803 (Figure 5B). In addition, after silencing MAP4K4; the expression of MAP4K4 was downregulated in MKN-28 and SGC-7901 cell lines compared with the control (Figure 5C,D). Meanwhile, the number of cells after the silencing of MAP4K4 was also reduced compared to the control group (Figure 5E,F). We only selected the shRNA1 to conduct the experiments because the shRNA1 with a better performance of silencing was easy to conduct in the process of the experiments. Thus, we omitted the experiments of shRNA2 in 5E/5F of the old manuscript.

**Figure 6:**
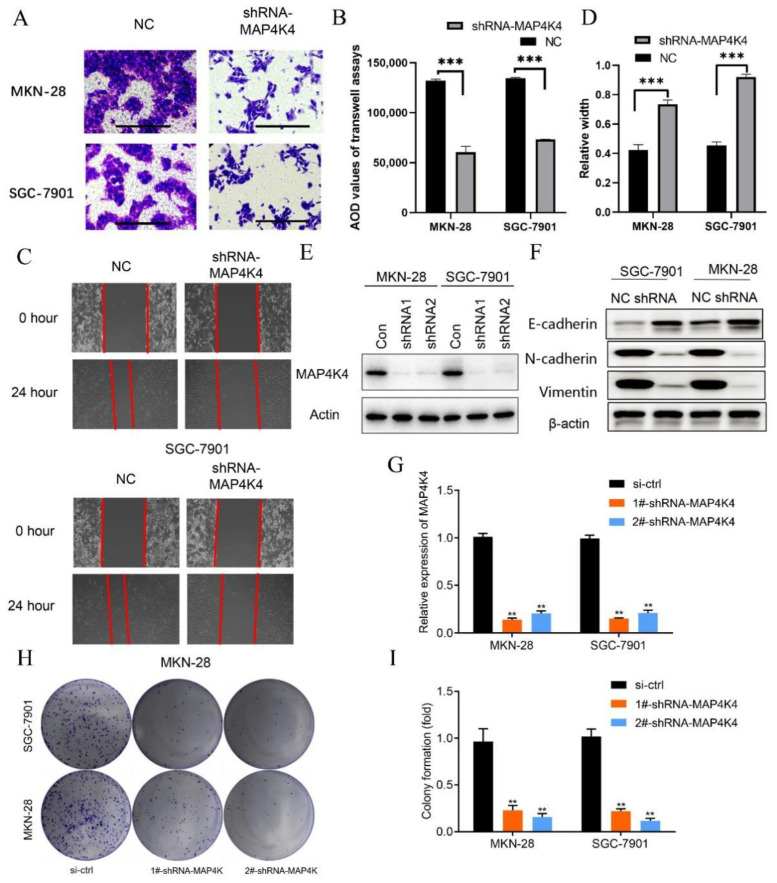
Detection of cell proliferation, invasion, and metastasis ability. (**A**) Transwell assay of normal condition (NC) and silenced MAP4K4 (shRNA-MAP4K4) in MKN-28 and SGC-7901 cell lines; (**B**) quantification from (**A**) the two-independent t-tests analyzes the difference of Transwell pictures’ AOD values; (**C**) wound-healing assay of normal condition (NC) and silenced MAP4K4 (shRNA-MAP4K4) in MKN-28 and SGC-7901 cell lines from 0 h to 24 h. (shRNA is delivered using a vector); (**D**) relative width of normal condition (NC) and silenced MAP4K4 (shRNA-MAP4K4) in MKN-28 and SGC-7901 cell lines in 24 h compared to 0 h. The two-independent t-tests analyze the difference in relative widths; (**E**) expression of MAP4K4/Actin determined by Western blot (WB) in MKN-28 and SGC-7901 cell lines before silencing MAP4K4 and after silencing MAP4K4 actin; (**F**) expressions of E-cadherin (Cat: AF748), N-cadherin (Cat: 13-2100), Vimentin (PRO-309), and control β-actin proteins determined by WB analysis in MKN-28 and SGC-7901 cell lines before silencing MAP4K4 (NC) and after silencing MAP4K4 (shRNA); (**G**) relative expression of MAP4K4 with si-ctl or silenced MAP4K4 (shRNA-MAP4K4) in MKN-28 and SGC-7901 cell lines; (**H**) clone formation assay of MKN-28 and SGC-7901 cells with si-ctl or silenced MAP4K4 (shRNA-MAP4K4); (**I**) clone formation statistics of MKN-28 and SGC-7901 cells with si-ctl or silenced MAP4K4 (shRNA-MAP4K4). Data of the three independent experiments are represented as mean ± SD. The significance is determined by two-way ANOVA. ** *p* < 0.01, *** *p* < 0.001. Correlational analyses are used to obtain the significant differences (Figure 6G,I).

Finally, it was observed that invasive and metastatic abilities were decreased in MKN-28 and SGC-7901 cell lines after MAP4k4 silencing (Figure 6A–D). Based on the Western blotting analysis, in MKN-28 and SGC-7901 cell lines, the expression of MAP4K4 was significantly decreased after silencing MAP4K4 compared with the control group (Figure 6E,G). The decreased N-cadherin and Vimentin protein expression indicated that the ability of the epithelial–mesenchymal transition (EMT) of cancer cells was also weakened (Figure 6F). The results of clone formation assays reveal that MKN-28 and SGC-7901 cell lines exhibit a significantly decreased ability of clone formation, and the number of clones is significantly decreased after MAP4K4 silencing compared with the control group (Figure 6H, I).

## 4. Discussion

Previous studies have shown that MAP4K4 plays multiple roles in cancer metastasis, and MAP4K4 can promote oncogenic transformation by regulating its downstream targets [24]. According to a recent study, mitogen-activated protein kinase isoform 4 (MAP4K4) is majorly involved in cancer cell growth [45]. Although there are some reports on the role of MAP4K4 in gastric cancer [27,45], complete and comprehensive bioinformatics and experimental analyses are still required to explore the entire role of MAP4K4. In the present study, we comprehensively analyzed the expression and regulatory mechanisms of MAP4K4 based on the gastric cancer public dataset obtained from TCGA. The overall analyses revealed MAP4K4 as a new and reliable potential marker that could predict the prognosis of gastric cancer.

TCGA analysis revealed that MAP4K4 expression in the tumor was significantly higher than in the adjacent samples, indicating that MAP4K4 might be a tumor-promoting gene in gastric cancer, which was consistent with the previous reports on cancers [20,21,22,23]. Moreover, the high-MAP4K4-expression group had a poor prognosis, indicating that MAP4K4 was associated with a poor prognosis. In the current study, the analysis also predicted that miR-582-3p, miR-141-3p, and miR-181c-5p could target MAP4K4. Therefore, the above results confirm and increase the reliability of the present study. The miR-582-3p/miR-141-3p/miR-181c-5p and MAP4K4 signaling mechanisms might also play a role in gastric cancer. In addition to the above miRNAs, we observed that miRNAs miR-96-5p and miR-29c-3p from the previous signaling pathways were also predicted by two databases in our research [27].

The Western blotting assay revealed increased E-cadherin protein expression after MAP4K4 silencing. In contrast, the expressions of N-cadherin and Vimentin proteins was decreased, indicating the lower EMT ability of cancer cells. Additionally, WB analysis determined the expression of E-cadherin, N-cadherin, Vimentin, and control β-actin proteins in MKN-28 and SGC-7901 cell lines (Figure 6D). It has been reported that miR-141-3p would promote the EMT process by targeting MAP4K4 [46], and MAP4K4 could promote EMT reprogramming in glioblastomas [23]. This was concordant with our experiments, indicating that MAP4K4 may also reduce adhesion and increase cell spreading.

MAP4K family kinases play important roles in immune cell signaling, immune responses, and inflammation [47]. MAP4K4 can be phosphorylated to induce TRAF2 protein degradation, leading to the negative regulation of IL-6 production in resting T cells [48]. In the present study, the protein interaction network of MAP4K4 revealed its interaction with TRAF2. The potential binding sites of miR-96-5p and miR-29c-3p on MAP4K4, and TLL_1_ mRNAs were reported in a previous study [27].

Although this study analyzed the role of MAP4K4 in gastric cancer, extensive research is needed to further evaluate the specific mechanism of MAP4K4 promoting the occurrence of gastric cancer. Recent evidence has suggested that TNF-α can activate MAP3K family member MLK3, which is essential for downstream JNK signaling activation [49]. MLK3 has been implicated in the progression of several cancers and plays a central role in cell survival and proliferation [50,51]. MLK3 is highly selective and interacts with multiple effectors. For instance, MLK3 can regulate the Ste20 family member Pak1 kinase and enhance tumor cell proliferation [52]. Since MAP4K4 also belongs to the Ste20 family and is activated by TNFα to participate in the JNK signaling pathway [25,49], we hypothesized that MAP4K4 may act upstream of MLK3 to promote tumor development. In addition, the analysis of the current study also needs further experimental verification.

In conclusion, MAP4K4 was identified as one of the overexpressed molecules in gastric cancer tissues and was closely related to the progression, invasion, and metastasis of gastric cancer. MAP4K4 high expression negatively impacts the prognosis of gastric cancer patients, suggesting that MAP4K4 should be regarded as an important prognostic factor for gastric cancer. Further attention should be paid to the abnormality of MAP4K4 in the progression of gastric cancer. We hope these results will spur the development of clinical trials and improve the survival rate of gastric cancer patients.

## 5. Conclusions

Collectively, our findings reveal that MAP4K4 plays a crucial role in the progression of gastric cancer. MAP4K4 is highly expressed in gastric cancer, predicting a poor prognosis and promoting gastric cancer invasion and metastasis. The obtained results provide a better understanding of the role of MAP4K4 in gastric cancer progression and a potential therapeutic target and prognostic predictor against this malignancy. Extensive research is needed to evaluate its effectiveness and to better understand the intricacies of its application.

## Figures and Tables

**Table 1:** Demographic and laboratory parameters of 21 gastric cancer patients included in the study.

Features	Cases
Total	21
Age median (range)	63 (33–80)
Gender	
Male	15 (71.4%)
Female	6 (28.6%)
Age	
≥60	15 (71.4%)
<60	6 (28.6%)
Lymph node metastasis	
Yes	17 (81.0%)
No	4 (19.0%)
Degree of differentiation	
I + II	5 (23.8%)
III + IV	16 (76.2%)

**Table 2:** All primer sequences of genes.

Gene Names	Forward Primer	Reverse Primer
MAP4K4	5′-TCTTTGGTCTTGTGGCATTA-3′	5′-GCCTTTCATTTGGCTGAT-3′
β-actin	5′-CCTAGAAGCATTTGCGGTGG-3′	5′-GAGCTACGAGCTGCCTGACG-3′

**Table 3:** Chi-squared-test analysis of clinical data and MAP4K4 expression.

Characteristics	High Expression (187)	Low Expression (188)	X^2^	*p*-Value
**Age**			1.7502	4.17 × 10^−1^
** <60**	50	62		
** ** **≥60**	135	124		
** Unknown**	2	2		
**Sex**			1.3153	2.51 × 10^−1^
** Female**	61	73		
** Male**	126	115		
**Pathologic_Stage**			4.2773	3.70 × 10^−1^
** Stage I**	27	26		
** Stage II**	47	64		
** Stage III**	80	70		
** Stage IV**	22	16		
** Unspecified**	11	12		
**T_Stage**			2.3766	6.67 × 10^−1^
** T1**	9	10		
** T2**	36	44		
** T3**	91	77		
** T4**	47	53		
** Unspecified**	4	4		
**N_Stage**			7.0953	1.31 × 10^−1^
** N0**	47	64		
** N1**	45	52		
** N2**	45	31		
** N3**	42	32		
** Unspecified**	8	9		
**M_Stage**			2.1004	3.50 × 10^−1^
** M0**	160	170		
** M1**	15	10		
** Unknown**	12	8		
**Race**			4.0659	3.97 × 10^−1^
** Asian**	37	37		
** Black**	5	6		
** Hawaiian**	0	1		
** Unknown**	20	31		
** White**	125	113		
**Histologic_Grade**			2.8209	4.20 × 10^−1^
** G1**	7	3		
** G2**	66	71		
** G3**	108	111		
** GX**	6	3		

## Data Availability

Not applicable.

## Abbreviations

CNVs	Copy number variations
CPM	Counts per million reads
EMT	Epithelial–mesenchymal transition
ESTIMATE	Estimation of STromal and Immune cells in MAlignant Tumor tissues using Expression data
GSEA	Gene-set-enrichment analysis
GO	Gene ontology
KEGG	Kyoto Encyclopedia of Genes and Genomes
MAP4K4	Mitogen-activated protein kinase kinase kinase kinase 4
SNVs	Single nucleotide variations
STAD	Stomach adenocarcinoma
TAMs	Tumor-associated macrophages
TCGA	The Cancer Genome Atlas
TNBC	Triple-negative breast cancer
TME	Tumor microenvironment
WB	Western blotting
